# Assessment of the transport management systems for National Acquired Immunodeficiency Syndrome (AIDS) Council of Zimbabwe Global Fund Round 8 grant sub-recipients and implementing partners, 2014

**DOI:** 10.11604/pamj.2020.37.226.21202

**Published:** 2020-11-09

**Authors:** Richard Makurumidze, Gombe Notion Tafara, Magure Tapiwa, Lucia Takundwa, Mufuta Tshimanga

**Affiliations:** 1 Department of Community Medicine, College of Health Sciences, University of Zimbabwe, Harare, Zimbabwe,; 2 National AIDS Council of Zimbabwe, Harare, Zimbabwe

**Keywords:** Transport management, donor, Global Fund, HIV & AIDS

## Abstract

**Introduction:**

during a Global Fund sub-sub recipients (SSRs) and implementing partners (IPs) review meeting for quarter 14 held in September 2013, several reports on mismanagement of vehicles were reported. We were then prompted to assess the transport management systems for the SSRs and IPs.

**Methods:**

we conducted a descriptive cross-sectional study. The study participants were managers, drivers and other personnel involved in transport management. We also assessed the conditions of the vehicles. Data were collected using a questionnaire and checklist.

**Results:**

we interviewed ten participants, seven from the IPs and three from the SSRs. Understanding and knowledge on the contents of the Memorandum of Understanding (MOU) which accompanied the vehicles were low. Six out of the ten organisations had operational vehicle policies but had shallow content. Eighteen (18) vehicles were assessed, 16 runners and two non-runners. Fifteen (15/18) of the vehicles did not have valid Zimbabwe National Authority for Road Administration (ZINARA) license discs. Only one (1/18) vehicle had a valid Zimbabwe Broadcasting Cooperation (ZBC) license disc. Of the 18 vehicles, 12 were insured with comprehensive insurance cover. Seven (7/18) of the vehicles were once involved in an accident. All the vehicles were serviced on a quarterly basis. Six (6/18) vehicles had both records of monthly service expenses and fuel returns. All the vehicles had logbooks, but only 8/18 of them were carbonated. Some sections of logbooks were incomplete.

**Conclusion:**

the transport management systems for the IPs and SSRs were below standard. We recommended the training and capacity building of IPs and SSRs in transport management.

## Introduction

Zimbabwe is among the countries in sub-Saharan Africa that have registered a dramatic decline in the burden of HIV. The number of new infections has decreased by 35,3% from 62,000 [45,000-83,000] in 2010 to 38,000 [28,000-51,000] in 2018 while the incidence has almost halved from 5.65 [4.08-7.67] to 2.79 [2.01-3.78] during the same period [1]. HIV related deaths have decreased by 42,6% from 54,000 [43,000-68,000] to 22,000 [17,000-27,000] between 2010 and 2018 [1]. These impressive health gains are attributed to behavioural and biomedical prevention interventions that have been implemented over the past several years as well as the widespread use of lifesaving antiretroviral therapy. The number of clients on antiretroviral therapy has gradually risen with more than 85% of people living with HIV on ART by the end of 2018 [2].

The cost of implementing the antiretroviral therapy (ART) programme has been a serious challenge in Zimbabwe with 78% of the cost currently being supported by external donors [3]. This scenario has further been exacerbated by the socio-economic crisis that the country has been subjected to for several years. Given this situation, the National AIDS Trust Funds (NATF) was introduced in 1999 as a domestic source of funding through the AIDS levy. The AIDS levy entails a 3% income tax for individuals and 3% tax on profits of employers and trusts (which excluded the mining industry until 2015) [4]. The funds are managed by the National AIDS Council (NAC) of Zimbabwe, a parastatal established for the coordination of multi-sectoral control of HIV within the country [5]. Since inception, the AIDS levy has been a critical domestic funding source for HIV, but it has fallen far short of financial needs of the national needs plans thereby resulting in overdependence on external support to meet the cost of the program [6,7]. Among the external funders of the ART program in Zimbabwe key is the Global Fund for AIDS, tuberculosis (TB) and Malaria (GFATM) which was by the end of 2018 contributing close to half of the cost of antiretroviral drugs in the country [8].

GFATM is a financing mechanism, a tool to channel large sums of money to programs fighting the three following communicable diseases; AIDS, TB and Malaria. It was established in January 2002 as a culmination of the hopes and hard work of people around the world from civic society and governments, north and south, each motivated by a belief that the three epidemics of AIDS, TB and Malaria could be reversed and their devastation stemmed [9]. To date, 32 million lives have been saved globally through this initiative [10]. It is a performance-based funding agency. On an annual basis, the GFATM raises and disburses nearly USD 4 billion to support programs in more than 140 countries [11]. Zimbabwe has periodically applied and received GFATM grants to support the antiretroviral therapy (ART) program. The grants awarded to date include HIV Round 1 (2002), 5 (2008) and 8 (2008), New Funding Model (NFM) (2014) and the joined TB/HIV (2017) currently being implemented [11-15].

The GFATM HIV Round 8 was implemented in 2 phases. Phase I was implemented from January 2010 to December 2011 and Phase II started in January 2012 and ended on the 31^st^ of December 2013. In the HIV Round 8 grant, the United Nations Population Development Fund (UNDP) was the principal recipient (PR) and NAC was a sub-recipient (SR). NAC had six sub-sub recipients (SSRs) and ten implementing partners (IPs). To improve the efficiency and effectiveness of the IPs and SSRs they were issued with vehicles and all received Toyota Hilux Double Cab 2.5 TDI trucks. In total, 20 vehicles were allocated when the grant started in 2010. The vehicles were issued through the PR accompanied by a Memorandum of Understanding (MOU), “memorandum of acceptance of temporary custody of UNDP property [vehicle]” [16].

During a SSRs and IPs review meeting for quarter 14 held in September 2013, several reports on mismanagement of vehicles were raised. A report on a vehicle from one IP which had broken down due to clutch plate damage was made. A quotation to repair the vehicle from Toyota, Zimbabwe, was around 12,000 USD. The vehicle mileage was close to 200,000 km while other vehicles which started operating at the same time had mileages less than 50,000 km. A vehicle from another IP from Mashonaland West Province was reported to be attached to messenger of court in Chinhoyi due to debt. Another IP from Masvingo Province was yet to send their accident damaged vehicle for repair. During the same meeting the NAC internal audit reported; five vehicles with incompletely filled log sheets, vehicles with unsanctioned trips, three vehicles seen travelling for unofficial business during weekends and after working hours, vehicles failing to pass police roadblocks and tollgates due to absence of necessary licenses and registration. The NAC management and the PR strongly felt that they could be transport mismanagement by the SSRs and IPs. We were then prompted to assess the transport management systems of the SSRs and IPs in line with the expectations of the MOU.

## Methods

We conducted a descriptive cross-sectional study. The study participants were the program managers, finance and administrative managers, drivers and other personnel involved in transport management. Data were collected using an interviewer-administered questionnaire with questions to assess the organisation transport management systems and policies. Inspections using a checklist were used to assess the motor vehicles. Data collecting tools were pre-tested using managers and drivers based in Harare at NAC headquarters and Harare Province NAC office. The data collection tools were then adjusted according to appropriateness, timing, interpretation, sequence and wording of questions. The data were analysed with Epi Info 3.5.1 using descriptive statistics. The permission to conduct the study was sought from the Ministry of Health and Child Care (MoHCC), Health Studies Office; chief executive officer for the National AIDS Council and the management of the visited IPs and SSRs. Consent was obtained from every participant prior to entry into the study. No names were written on the data collection tools. Confidentiality was assured and maintained.

## Results

Ten organisations, seven IPs and three SSRs were visited during the assessment. From each organisation, an individual with either management or administrative role who understood the transport management system, policies and practices of the organisation was interviewed. Out of the ten participants; four were programme managers, four were finance and administrative managers and the other two belonged to other professions. Six were males and four were females and the median age in years was 44 interquartile range (IQR) (35-48). The median duration in service in years was 7 IQR (4-8).

The IPs and SSRs on receiving the vehicle were given the document “memorandum of acceptance of temporary custody of UNDP property [vehicle] (MOU)” which outlined the transport requirements by the PR. All the interviewed ten managers mentioned that they had seen the document, but when they were asked to produce the document none of them did. Five of the managers mentioned that they had read the MOU. The five managers who mentioned that they read the MOU were asked to mention the transport management requirements in the document. The managers struggled to mention the components. None of the managers mentioned that the SSR or IP had to cover the expenditure of vehicle and third-party insurance claims. The most mentioned component was that the vehicle was a UNDP property and was mentioned three times.

All the organisations had focal persons responsible for transport management. In five of the organisations, the transport focal person was the finance and administrative manager. Only three of the organisations had someone qualified to manage transport. The three had a transport officer, fleet manager and logistic manager. Nine of the organisations had a transport request form and in eight of the organisations, the programme manager was responsible for authorising transport requests. Of the ten organisations, six had vehicle operational policies and all were seen and read. The contents of the six policies were analysed. All the policies had the components on the acquisition and disposal of vehicles. None of the policies had the following components; change of tyres, vehicle insurance and licenses, insurance for employees and training of drivers.

A total of 18 vehicles were assessed, of which 16 were runners and two were non-runners. The vehicles were assessed for the period between 2010 when the IPs and SSRs received the vehicles to the 31^st^ of May 2014. Ten of the vehicles had their registration books available while the other eight had their registration books still with MoHCC. Most of the vehicles were parked at the organisation premises while four were parked at a designated parking area. Fifteen of the vehicles did not have a valid Zimbabwe National Authority for Road Administration (ZINARA) license discs. Only one vehicle had a valid Zimbabwe Broadcasting Cooperation (ZBC) license disc. Of the 18 vehicles, 12 were insured with comprehensive insurance cover. The average insurance cover cost for the 12 insured vehicles was 26,600 USD (standard deviation ± 400 USD). Four of the vehicles were registered in the name of MoHCC and were exempted from paying ZINARA.

Seven of the vehicles were once involved in an accident during the period of assessment. Six of the accidents were road traffic accidents and one occurred when the driver left the vehicle parked on a steep descent while it was raining. All the seven accidents were reported, but only three vehicles had their accident reports seen. Only one vehicle had a serious mechanical fault. This was clutch plate damage and the fault was reported to the authorities. The SSRs and IPs were asked on the procedure in case an employee is involved in an accident. All of them mentioned the need to have a police report, employee report, informing the insurance and funder and conducting a body of enquiry. Only one organisation mentioned that they visit the scene of the accident. Only one organisation also mentioned that an employee might be asked to cover the cost of repair in case there is a deficit from the insurance and the employee was found guilty. Four organisations mentioned that they suspend the drivers if the body of inquiry found that the accident was due to negligence.

Most of the vehicles (14/18) were serviced at Toyota Zimbabwe, while the other four were serviced at other garages. The proof of servicing was available for 14 vehicles. All the vehicles were serviced on a quarterly basis and servicing was not based on schedule but on availability of funds. None of the vehicles had a schedule for future servicing. Most of the vehicles had their accessories available except a few which had missing screwdrivers (1/18), spanners (2/18) and pliers (3/18). Only two vehicles had spare keys.

Of the 18 vehicles, only eight had both monthly service and repair and fuel returns. The average monthly fuel return for the eight vehicles was 10.25 km per litre of fuel. Using an average consumption of between eight litres of fuel per kilometre, 6/18 of the vehicles had normal consumption. Seven of the organisations highlighted that they use the monthly vehicle return information. The most mention use was assessing vehicle usage (6/10), guidance on fuel requirements and fuel accountability (5/10) and checking vehicle performance (3/10). All the vehicles had logbooks, but only 8/18 were carbonated. Two of the logbooks had missing mileages, i.e. not all mileages were recorded and distance travelled was not accounted for. In all the assessed log books date, departure time, arrival time and initial mileage were filled. Five (5/18) logbooks had incomplete sections. In five of the logbooks, the purpose of the trip was not mentioned and in four, the destination was not indicated. Six (6/18) of the logbooks had trips recorded after working hours of the organisation.

We assessed a total of 12 drivers, eight males and four females. The median age of the drivers in years was 34 IQR (31-44.5). The median driving experience in years was 11 IQR (6-14). Most of the drivers (8/12) were not strictly drivers but officers who had other duties and responsibilities on top of driving. Only 4/12 of the drivers had a written authority to drive Global Fund vehicles while the rest had verbal agreements. Only four drivers had valid defensive driving licenses. We assessed the drivers' knowledge on items that should be checked before driving a vehicle, vehicle servicing and logbook. All the drivers knew that a vehicle should be serviced after at least 5,000 km. All the drivers mentioned that it was their responsibility to complete the logbook. However, 10/12 mentioned that at times they forgot to fill the logbook. When asked on what items are supposed to be filled in a logbook, all of them were aware.

## Discussion

The assessment showed that the transport management system for SSRs and IPs was below standard. Majority of the organisations did not have the MOU from the PR, i.e. UNDP and transport management policies. Most of the vehicles did not have the required licenses, were not being insured on time and did not have monthly vehicle returns. Though most of the vehicles had logbooks, they were poorly completed and not carbonated. Majority of the drivers did not have defensive driving and written authority from the funder to drive the vehicles.

The non-availability of the UNDP MOU within the organisations was most likely responsible for the poor knowledge and understanding of the MOU contents. The MOU outlined the transport requirements. The non-availability might have been due to poor recording keeping and filing leading to misplacing, which was glaring during the interviews with most of the IPs and SSRs. From the non-availability and poor understanding of the MOU, one will conclude that the majority of SSRs and IPs were not managing their transport system according to the requirements of the PR. In most of these organisations, the finance and administrative manager was responsible for managing the transport system. Finance and administrative managers are not the best people to manage transport system since they have a lot of other responsibilities. The majority have none or little knowledge of transport management since it is not part of their primary training. The reason why the other organisations were unable to employ qualified people might have been due to the small size of the organisations and budgetary constraints [17].

The assessed vehicles management policies lacked the critical areas required in a vehicle policy. The organisations might have avoided putting some of the items in their policies due to the cost that will be incurred if those areas were part of the policies. The other reason might have been due to lack of knowledge and failure to read and follow the transport requirements in the MOU from the PR. Surprisingly, all the policies assessed included the acquisition and disposal of vehicles. The management of these organisations might have added that with a hidden benefiting agenda. Generally, a vehicle policy should be detailed and should cover the following issues: who should be allowed to drive the vehicles; vehicle utilisation; maintenance and servicing; accident reporting procedures; acquisition and disposal of vehicles; licenses and insurance among other things [18].

Most of the vehicles were not insured and licensed. There are several reasons why the vehicles were not insured and licensed. There was a delay in the disbursement of funds by the PR for the New Funding Model grant. The funds came in the 2^nd^ quarter of 2014, making it impossible to insure and license the vehicles when the year began. Most of the organisations due to budgetary constraints were unable to pre-fund since they solely depended on the Global Fund support for all their activities. The organisations might have also failed to pay licenses and insurances for the whole year since they receive their disbursements on a quarterly basis. Some vehicles were not insured or licensed because not all IPs and SSRs continued with the New Funding Model grant. Those IPs and SSRs which did not continue had their vehicles parked and not in use. The other reason was that some of SSRs and IPs were yet to get registration books for their vehicles from MoHCC. The registration books are required for the licensing and insuring processes. The vehicles which were registered in the MoHCC name were exempted from paying some of the licensee fees. This arrangement could be adopted to cushion other organisations with financial constraints.

Not all the organisations serviced the vehicles with the recommended service provider, Toyota Zimbabwe. This was because not all the IPs and SSRs had access to a Toyota Zimbabwe garage in their area. They were servicing vehicles at a garage within their vicinity to minimise cost. The other reason was that the PR requested three quotations before vehicles are serviced and the SSR or IP is supposed to choose the cheapest. The cheapest might not be Toyota and the vehicle will end up being serviced by a different service provider. Servicing cars by different service providers is not encouraged since there is no continuity leading to loss of service history. The organisations serviced their vehicles on a quarterly basis and were not timely but based on the availability of funds. The findings are consistent with a study by Masoja *et al*. which showed that in some cases, organisations fail to services vehicles routinely due to inadequate budgetary allocations [19]. Maintenance and servicing of vehicles are crucial for the sustainability of vehicles within an organisation. Servicing is a preventive measure that ensures that potential problems are identified and dealt with promptly before the vehicle is damaged. Servicing is cheaper and allows cars to last longer on the road. Failure to service vehicles is usually associated with incurring huge cost later in case the vehicle breaks down [20,21].

Only one organisation mentioned that they visit the scene in case an employee is involved in an accident. The practice might not be practical because it is time-consuming and the accident might occur in the field, kilometres away from the office. The practice is, however, encouraged because it will enable those who conduct the disciplinary hearing to understand the context of the driver during the accident. Suspending the employee and making the employee cover the cost of repairing vehicle were mentioned as accident handling procedures. Though de-motivating, the two can be implemented within an organisation to act as deterrents. Not all the organisations performed monthly vehicle returns on their vehicles. This might have been attributed to lack of knowledge and awareness of the importance and purpose of monthly vehicle returns. Vehicle monthly returns help to assess vehicle utilisation, performance and efficiency [22].

Most of the vehicle logbooks were not carbonated. Carbonated logbooks make it is easier for the organisation to perform monthly vehicle returns. The used pages can easily be pulled off and sent for assessment by managers on a monthly basis. Some vehicles had trips recorded after offices hours and using that information; it might be incorrect to conclude that the concerned vehicles are being used for other purposes after hours. In most circumstances, field activities may go beyond office hours depending on the distance back to the office. Vehicle usage within an organisation should have proper guidelines to avoid abuse of the vehicles since other studies have shown that vehicles are often used for private and personal activities [23,24]. The missing mileages in the logbooks might point towards serious misuse of vehicles. The missing mileages are the distance of kilometres that will absent in the logbook and are unaccounted for. The driver will be skipping some kilometres as fill they logbook.

Only a few of the drivers had valid defensive driving training. This might have been because the defensive driving license expires every year and training is centralised making access difficulty for IPs and SSRs in the peripheries. Defensive driving is necessary, especially for drivers involved in driving long distances, carrying passengers and bad terrain roads, circumstances common among IPs and SSRs. Most of the drivers had no written authority to drive Global Fund vehicles. Giving someone written authority to drive minimises vehicle abuse. The drivers forgot to fill and complete the logbooks. The finding was similar from a study done in Mashonaland West Province, which showed that at times drivers forgot to complete the logbook [24]. The forgetting might be due to the pressure often associated with fieldwork. The finding confirmed the result that the majority of the drivers were not strictly drivers but officers who had other duties and responsibilities on top of driving.

## Conclusion

The majority of the IPs and SSRs had transport management systems which were below standard. From our study findings, we recommended the following: re-issuing all the IPs and SSRs with the “memorandum of acceptance of temporary custody of UNDP property [vehicle] (MOU)” document; training and capacity building in transport management and designing of operational vehicle policies; collection of vehicle registration books still with MoHCC on behalf of IPs and SSRs; all vehicles logbooks be carbonated; defensive driving training and written authority for the drivers.

### What is known about this topic

- Most of the vehicles which support the implementation of health programs in low resource settings are donor-funded;
- The area of how health programs vehicles are managed is overlooked;
- There is a paucity of literature in the public domain on how these vehicles are managed.

### What this study adds

- The study is among a few to investigate how vehicles within donor-funded programs are being managed;
- The findings show that there might be serious gaps in the capacity of organisations in low resource settings in managing vehicles they receive from donors;
- There is a need for capacity building in the area of vehicle and transport management in low-resource settings for sustainability.
